# Cytotoxic and apoptogenic effects of *Bryonia aspera* root extract against Hela and HN-5 cancer cell lines

**Published:** 2017

**Authors:** Solmaz Pourgonabadi, Mohammad Sadegh Amiri, Seyed Hadi Mousavi

**Affiliations:** 1 *Department of Pharmacology, School of medicine, Mashhad University of Medical Sciences, Mashhad, Iran*; 2 *Department of Biology, Payame Noor University, 193953697- Tehran, Iran*; 3 *Pharmacological Research Center of Medicinal Plants, School of Medicine, Mashhad University of Medical Sciences, Mashhad, Iran*; 4 *Medical Toxicology Research Center, Mashhad University of Medical Sciences, Mashhad, Iran*

**Keywords:** Bryonia aspera, Cytotoxicity, Apoptosis, Cancer

## Abstract

**Objective:**

*Bryonia aspera* (Stev. ex Ledeb) is a plant that grows in northeast of Iran. In the present study, cytotoxic and apoptogenic properties of *B. aspera* root extract was determined against HN-5(head and neck squamous cell carcinoma) and Hela (cervix adenocarcinoma) cell lines.

**Materials and Methods:**

HN-5 and Hela cell lines were cultured in DMEM medium and incubated with different concentrations of *B. aspera* root extract. Cell viability was quantitated by MTT assay and the optical absorbance was measured at 570 nm (620 nm as the reference) by an ELISA reader, in each experiment. Apoptotic cells were assessed using PI staining of DNA fragmentation by flow cytometry (sub-G1 peak). The *B. aspera* inhibited 50% growth (IC50) of Hela and HN-5 cell lines at 100±28 μg/ml and 12.5±4 μg/ml, respectively after 48 hr of incubation.

**Results:**

Cell viability assay showed that inhibitory effects of *B. aspera* were time and dose-dependent in both cell lines, which were consistent with morphological changes, observed under light microscope. Apoptosis was investigated by flow cytometry in which percentage of apoptotic cells increased in a dose and time-dependent manner.

**Conclusion:**

Based on our data,* B. aspera* has cytotoxic effects in which apoptosis played an important role. Further evaluations are needed to assess the possible anti-tumor properties of this plant.

## Introduction

Cancer, which has restricted efficient therapies, is one of the leading causes of death (Hsiao and Liu 2010 ▶). As the most of morbid of human cancer, head and neck squamous cell carcinoma is the eighth common cancer disease in the world (Stewart and Kleihues 2003 ▶; Forastiere et al 2001 ▶). HN-5 cells was derived from head and neck squamous carcinoma. Annually, this type of cancer affects approximately 600,000 patients worldwide. Standard treatment strategies include surgery, radiotherapy and chemotherapy. For treatment, several chemotherapeutic agents have been used and in 30–40% of patient-chemotherapy regimens have shown positive responses (Forastiere et al 2001 ▶; Parkin et al 2002 ▶). Worldwide, the second most frequent malignant tumor in women is cervical adenocarcinoma cancer. The cell line was derived from cervical cancer cells taken from Henrietta Lacks, in 1951. Hela cells are human epithelial cells from a fatal cervical carcinoma (Tabrizi et al 2006 ▶; Farjadian et al 2003 ▶). Considering significant levels of toxicity and drug resistance of current anticancer regimens, development of effective drugs with little or no adverse effects is crucial. Naturally occurring chemicals including plants derivatives provide a source of novel and potent bioactive compounds with minimal side effects (Cooper 2004 ▶; Tsao and Zeltzer 2005 ▶).

Herbal therapies and natural remedies are utilized all over the world and several drugs have been originated from herbs (Cooper 2004 ▶; Cooper 2005 ▶). 

*Bryonia aspera* Stev. ex Ledeb (Cucurbitaceae family) is native to Iran. This family has anti-inflammatory, anti-tumor, hepatoprotective, and immunomodulatory activities (Efferth et al 2001 ▶; Tsao and Zeltzer 2005 ▶). Cucurbit plants were recognized to have significant biological values. Ethnopharmacological information show that roots of *B. aspera *Stev. ex Ledeb, also known as “andaz”, have been traditionally used for treatment of gastrointestinal and cardiac diseases and cancer in the Turkmen Sahra region, north-east of Iran (Ghorbani 2005 ▶). 

Despite of these findings, the role of apoptosis in *B. aspera* induced toxicity has not been understood. Therefore, in this study the cytotoxic and apoptogenic effects of hydro-alcoholic extract of *B. aspera* against HN-5 and Hela cells were studied. 

## Materials and Methods

**Reagents and Chemicals**

Hela and HN-5 cell lines were obtained from Pasteur Institute (Tehran, Iran). Dulbecco’s modified Eagle’s Medium (DMEM), Penicillin-streptomycin solution and fetal calf serums (FCS) were purchased from Gibco (Grand Island, USA). The fluorescent probe propidium iodide (PI), sodium citrate, Triton X-100 and 3-(4, 5-dimethylthiazol-2- yl)-2, 5-diphenyl tetrazolium (MTT) were purchased from Sigma (St Louis, MO, USA). Dimethyl sulfoxide (DMSO) was bought from Merck (Darmstadt, Germany).

**Plant material**

The root of *B. aspera* Steven ex Ledeb. was collected from Tirgan Watershed, Razavi Khorasan Province, Iran. The Voucher specimen (No.21733) was deposited in Dargaz Payame Noor University Herbarium.

**Preparation of Extract**

*B. aspera* was identified by Pharmacological Research Center of Medicinal Plants. The roots extract was prepared from 20 g of dried and milledroots. The extract was prepared using 90 ml ethanol (70%) by Soxhlet apparatus. After that, the solvent was removed by evaporation at 36-37º C.

**Cell culture**

Cell lines were kept at 37°C in a humidified atmosphere (90%) containing 5% CO2. Cells were grown in Dulbecco's modified Eagle's medium (DMEM) with 10% fetal bovine serum, 100 U/ml penicillin, and 100 μg/ml streptomycin. Cells were seeded overnight, and then incubated with various concentrations of *B. aspera* root extract (12.5 to 500μg/ml) for 24, 48 and 72 hr. For MTT assay, cells were seeded at 5×10^3^ cell/well onto 96-well culture plates. For analysis of apoptosis, cells were seeded at 1×10^5^ cell/well onto a 24-well plate. For each concentration and time course study, there was a control sample, which stayed without extract and received the equal volume of medium. All experiments were performed in triplicate.

**Cell viability**

Cell viability was determined using a modified 3-(4, 5 dimethylthiazol-2-yl)-2, 5-diphenyl tetrazolium (MTT) assay (Mosmann, 1983 ▶; Mousavi et al, 2008 ▶; Mousavi et al, 2009 ▶; Sharifi et al, 2005 ▶).

In brief, cells were seeded at 5×10^3^/well onto flat-bottomed 96-well culture plates and allowed to grow for 24 hr followed by treatment with *B. aspera* root extract (12.5 to 500μg/ml). After removing the medium, MTT solution was added to cells (5 mg/ml in PBS) for 3 hr. The absorption was quantitated at 570 nm (620 nm as the reference) using an ELISA reader.

**Apoptosis**

Apoptotic cells were identified using PI staining (Mousavi et al 2008 ▶; Mousavi et al 2009 ▶; Sharifi et al 2005 ▶; Zhang et al 1999 ▶). Briefly, cell lines were cultured overnight in a 24-well plate and treated with *B. aspera* for 48 hr. Floating and adherent cells were cultured and incubated overnight at 4 °C in the dark with 750 μl of a hypotonic buffer (50 μg/ml PI in 0.1% sodium citrate plus 0.1% triton X-100). Then, apoptosis rate was measured by a FACScan flow cytometer (Becton Dickinson).

**Statistical analysis**

 One way analysis of variance (ANOVA) and Bonferroni’s *post hoc* were applied for data analysis. All results were expressed as mean±SEM and p<0.05 was considered as statistically significant.

## Results

**Effect of** ***B. aspera*** **on cell viability**

Hela and HN-5 cell lines were incubated with various concentrations of *B. aspera* root extract (12.5-500 μg/ml) for 24, 48 and 72 hr. Treatment with 50, 100 and 500 µg/ml of *B. aspera* root extract for 24hr, significantly decreased the percentage of viable Hela cells to 82.6±2.8 (p<0.05), 75.3±3.6 (p<0.01) and 60±1 (p<0.001), respectively. After 48hr incubation with concentrations of 12.5, 25, 50, 100, and 500 µg/ml of *B. aspera* root extract decreased viability to 99.7±4.2 (p<0.01), 72.8±7 (p<0.01), 72.76±4.05 (p<0.001), 48.9±3.8 (p<0.0001), 16.5±0.04 (p<0.0001), respectively.

**Figure 1:**
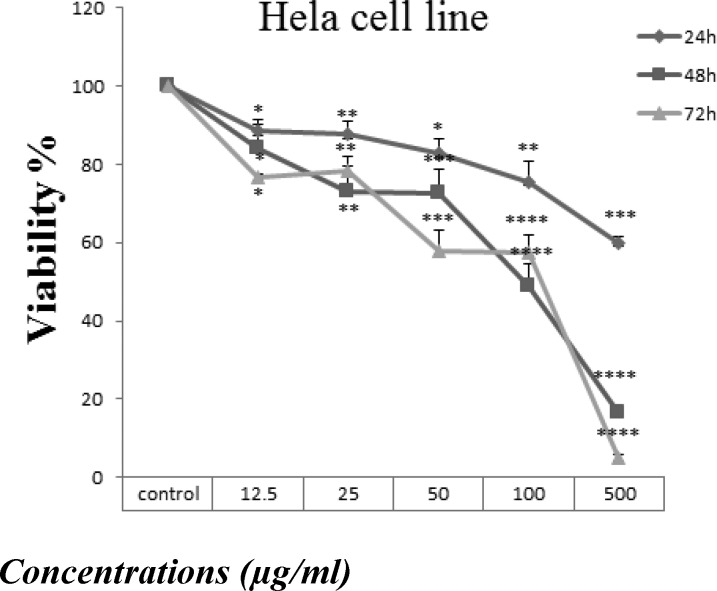
Effect of hydro-alcoholic *B. aspera* extract on viability of Hela (a), HN-5 (b) cells and normal cells (c). Cells were treated with different concentrations of *B. aspera* root extract for 24, 48 and 72 hr. Viability was quantitated by MTT assay. Results are expressed as mean ± SEM (n = 3). The asterisks are indicator of statistical differences obtained separately at different time points as compared to their control values (*p< 0.05, **p< 0.01, ***p< 0.001, ****p< 0.0001

After 72 hr of treatment with 12.5, 25, 50, 100 and 500 µg/ml of *B. aspera *root extract, the percentage of viable Hela cells declined to 76.77 ± 0.5 (p<0.05), 78.3 ± 0.9 (p<0.01), 58 ± 3.7 (p<0.001), 57.5± 3.1 (p<0.001) and 5.1 ± 0.38 (p<0.0001), respectively (Figure 1a). 

For HN-5 cell line, 24 hr of treatment with 12.5, 25, 50, 100 and 500 µg/ml of *B. aspera* root extract significantly decreased the percentage of viable cells to 99.6±4.08 (p<0.0001), 73.6±1.4 (p<0.0001), 68.2±3.2 (p<0.0001), 73.2±0.7 (p<0.0001) and 63±1 (p<0.0001), respectively and after 48 hr of incubation, viability decreased to 50±0.8 (p<0.0001), 40.5±3.2 (p<0.0001), 36.4±1.03 (p<0.0001), 40.2±0.18 (p<0.0001) and 29.5±1.4 (p<0.0001), respectively. After 72 hr treatment with 12.5, 25, 50, 100 and 500 µg/ml of *B. aspera* root extract the percentage of viable cells decreased to 56.8±0.66 (p<0.0001), 11.2±0.31 (p<0.0001), 7.3±0.17 (p<0.0001), 8±0.4 (p<0.0001) and 5.8±0.12 (p<0.0001), respectively, (Figure 1.b).

In normal cells, treatment with 12, 25, 50,100 and 500 µg/ml of B. aspera root extract for 24 hr did not decrease the percentage of viable cells. Treatment with 50, 100 and 500 µg/ml of B. aspera root extract for 48 hr decreased the percentage of viable cells to 69.21±3.38 (p<0.0001), 64.41±2.57 (p<0.0001) and 60.21±1.66 (p<0.0001), respectively. After 72 hr treatment with 25, 50, 100 and 500 µg/ml of B.aspera root extract the percentage of viable cells decreased to 78.92±7.47 (p<0.05), 42.45±2.77 (p<0.001), 40.56±2.28 (p<0.001) and 43.25±0.78 (p<0.001), respectively (Figure1c).

**Figure 2:**
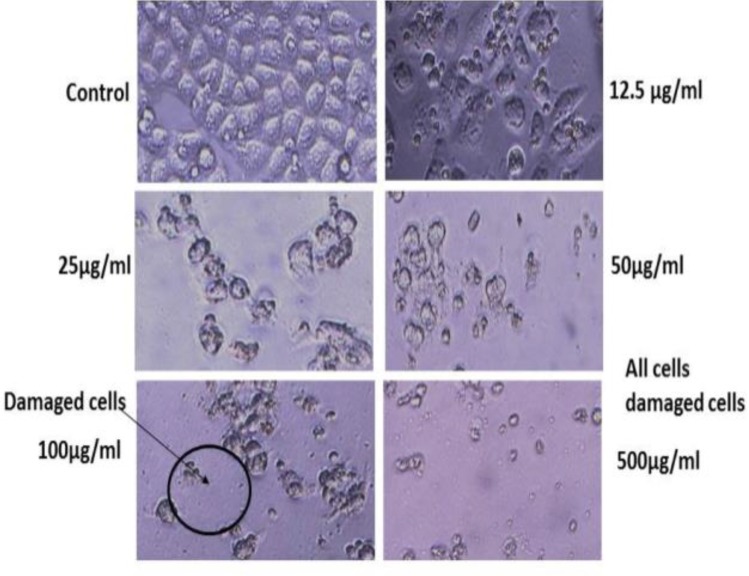
Effect of *B.* *aspera* (12.5-500µg/ml) on morphological changes in cultured cervix cancer (Hela cells) after incubation. Hela cells were completely damaged and round at 500µg/ml and partly at 100µg/ml. Morphologic changes included reduction in volume and rounding until the nucleus constituted the majority of the cellular volume

After incubation with *B. aspera* root extract, morphologic changes (reduction in volume and rounding until the nucleus constituted the majority of the cell volume) were observed in Hela cells (Figure 2).

As shown in Figures 1 and 2 *B. aspera* root extract decreased cell viability in Hela and HN-5 cell lines in a concentration and time-dependent manner.

**Role of apoptosis**

**Figure 3:**
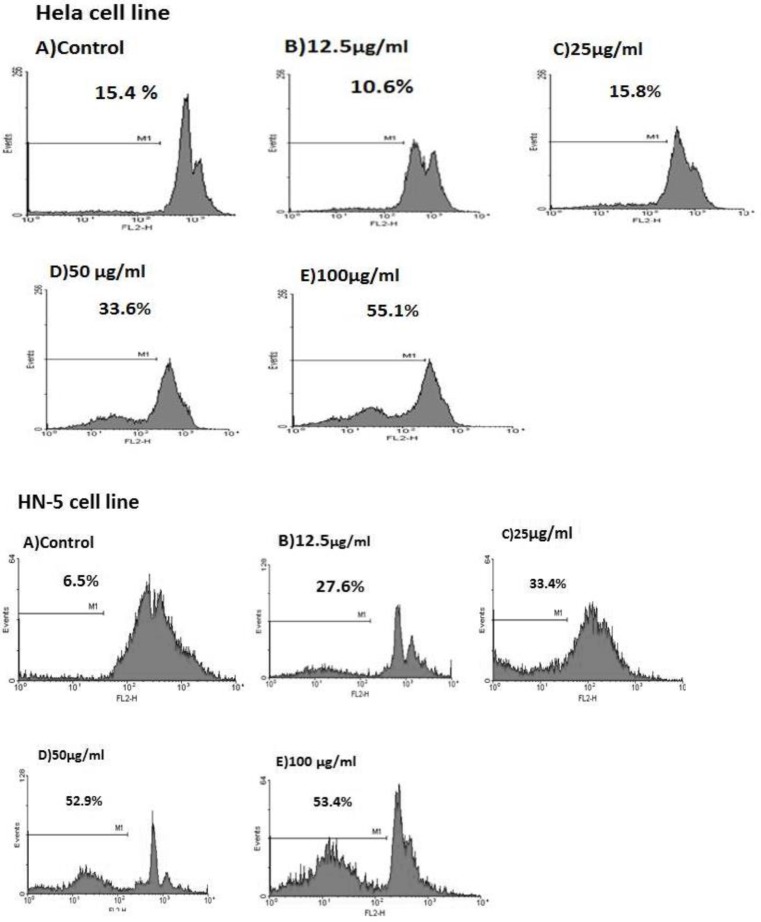
Flowcytometry histograms of apoptosis analysis following PI staining in Hela and HN-5 cell lines. Cells were treated with 12.5-100 μg/mL *B.* *aspera* root extract for 48 hr. Sub-G1 peak as an indicative of apoptotic cells, was induced in *B.* *aspera* root extract-treated cells

Sub-G1 peak is one of the reliable biochemical markers of apoptosis. There was a sub-G1 peak in flowcytometry histogram of *B. aspera*-treated but not in control cells indicating apoptotic cell death is involved in *B. aspera*-induced toxicity in Hela and HN-5 cell lines (Figure 3).

The rate of apoptosis induced by *B. aspera* in Hela and HN-5 cells is shown in Table 1.

**Table 1:** The rate of apoptosis in HN-5 and Hela cells following 48-hr treatment with *B.* *aspera* root extract

Apoptosis%	Control	12.5 µg/ml	25 µg/ml	50 µg/ml	100 µg/ml
Hela cell line	15.4	10.6	15.8	33.6	55.1
HN-5 cell line	6.5	27.6	33.4	52.9	53.4

## Discussion

Natural products have been utilized to prevent and treat many diseases including cancer, so they are good candidates for the development of anticancer drugs (Song et al, 2005 ▶). In vitro cell proliferation inhibition test using MTT viability assay confirmed that hydro-alcoholic root extract of *B. aspera* has cytotoxic activity against Hela and HN-5 cell lines. This data is consistent with previous study in which anti- proliferative effects of this extract were evaluated on MCF-7 (human breast adenocarcinoma), HepG2 (human hepato cellular carcinoma) and WEHI (mouse fibrosarcoma) cell lines (Sahranavard et al, 2010 ▶).

Here, the cytotoxic effects of *B. aspera* root extract and the role of apoptosis in this effect were studied for the first time. The results showed *B. aspera*–induced apoptosis was involved in induction of cell death. Apoptosis is a gene-regulated phenomenon, which is induced by many chemotherapeutic agents in cancer treatment (Song et al 2005 ▶). It is distinguished by distinct morphological features including chromatin condensation, cell and nuclear shrinkage, membrane blebbing, and oligonucleosomal DNA fragmentation (Hersey and Zhang, 2001 ▶). The induction of apoptosis in tumor cells is considered extremely beneficial in cancer therapy as well in the prevention of cancer. A variety of natural substances has been shown to have the ability to induce apoptosis in different types of cancer cells (Green and Reed, 1998 ▶).

The present study is the first to reveal cytotoxic effects of *B. aspera* on Hela and HN-5 cell lines in which apoptosis was involved. Further studies are required to determine the mechanisms involved in the cytotoxic activities of HN-5 and Hela cell line. *B. aspera* root extract could be considered as a potential chemotherapeutic agent in cancer treatment after further studies.
